# A Rare Case of Myxoid Adrenocortical Carcinoma

**DOI:** 10.7759/cureus.39433

**Published:** 2023-05-24

**Authors:** Carmela Claire Ferrer, Pamela R Delos Reyes-Murillo

**Affiliations:** 1 Pathology and Laboratory Medicine, National Kidney and Transplant Institute, Quezon City, PHL

**Keywords:** myxoid, myxoid adrenocortical carcinoma, adrenal cortex neoplasms, adrenal gland neoplasms, adrenocortical carcinoma (acc)

## Abstract

Myxoid adrenocortical carcinoma (myxoid ACC) is a rare subtype of adrenal cortical carcinoma with only a few cases reported in the literature. This tumor is characterized by small to large neoplastic cells in cords, diffuse sheets, or nodular architecture, which are surrounded by variable amounts of myxoid material. We are presented with an elderly female with a suprarenal mass which revealed a tumor composed of neoplastic cells surrounded by scant to abundant myxoid stroma. Expression for Melan-A, Inhibin, Synaptophysin, and Pancytokeratin, as well as a Ki-67 proliferative index of 15%, warrant a diagnosis of myxoid ACC.

## Introduction

Adrenocortical carcinoma (ACC) is a rare neoplasm affecting 0.7-2 per million cases in the United States, with a higher incidence rate for the female sex [1-2]. It commonly occurs during the fifth and sixth decades [1-2]. Unilateral involvement is common [1-2], and usually, the left adrenal gland is affected [2]. However, ACC may also occur in ectopic sites such as the retroperitoneum, pelvic (in between the urinary bladder and prostate), and ovary [2].

ACC can be categorized as functioning or non-functioning. About half of the population with ACC are diagnosed with functioning ACC, presenting with signs and symptoms of hormone secretion. A majority of patients with functional ACC may have signs and symptoms of glucocorticoid excess [1-2] or may present with signs and symptoms related to glucocorticoid and sex-steroid secretion [2]. On the other hand, a minority of patients with functional ACC present with features associated with sex-steroid secretion only [2]. Only a handful of functional ACC exhibit excess aldosterone-related signs and symptoms [1-2]. Symptoms associated with mass effects, such as abdominal and/or flank pain, are often seen in patients with non-functional ACC [1-2].

ACC macroscopically presents as a large, solitary tumor, often weighing more than 100 grams [2]. Serial sections of the tumor disclose a variegated, yellow-to-tan cut surface [1-2]. Heterogeneity and high-grade morphology of the tumor may be seen in areas of necrosis and hemorrhage [1-2]. Based on histomorphologic features, ACC can be classified into different subtypes such as conventional ACC, oncocytic ACC, myxoid ACC, and sarcomatoid ACC [1-2].

The neoplastic cells in conventional ACC are seen arranged in broad trabecular, solid, or nested growth patterns [2]. The neoplastic cells often exhibit diffuse growth, cytologic atypia, and eosinophilic cytoplasm [2]. Mitotic figures are frequently present [1-2]. For the diagnosis of oncocytic ACC, the tumor must be composed of >90% of oncocytic cells. Oncocytic cells are described as neoplastic cells with high-grade nuclei and abundant eosinophilic cytoplasm [2]. Sarcomatoid ACC is a rare biphasic tumor composed of a conventional ACC component and a sarcomatoid component [1-2]. However, a monophasic tumor is mostly composed of the sarcomatoid component [2]. The sarcomatoid component is characterized by spindle cells with nuclear pleomorphism [1]. Atypical mitotic figures and tumor giant cells may also be seen [1]. Judicious sampling must be done in order to differentiate a sarcomatoid ACC from a primary sarcoma [1]. Lastly, myxoid ACC is an uncommon subtype of ACC that is characterized by cords and trabeculae of small to large neoplastic cells embedded in a myxoid matrix [1-2]. Macroscopic features of this neoplasm are almost the same as with the other subtypes, but this tumor often exhibits gelatinous, translucent, myxoid areas [1]. Myxoid ACC is often under-reported due to its lack of diffuse growth and mild cytologic atypia [1-3].

This paper aims to suggest the necessity of using the accepted multifactorial scoring systems with the aid of immunohistochemical stains in order to diagnose rare adrenocortical neoplasms. Herein, we present a female in her 50s who presented with flank pain and suprarenal mass.

This case report was previously presented as a poster at the 72nd Philippine Society of Pathologists Annual Convention on April 21, 2023.

## Case presentation

**Figure 1:**
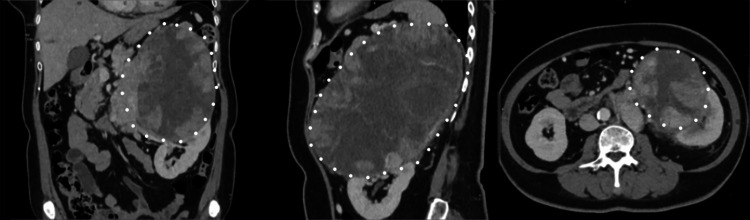
Contrast-enhanced whole abdominal CT scan Encircled in white: suprarenal mass

This is a case of a 54/F who presented with a history of flank pain. Triple phase computed tomography scan of the abdomen showed a 20.7 x 13.9 x 14.3 cm large, lobulated, enhancing mass in the left suprarenal region (Figure 1).

**Figure 2:**
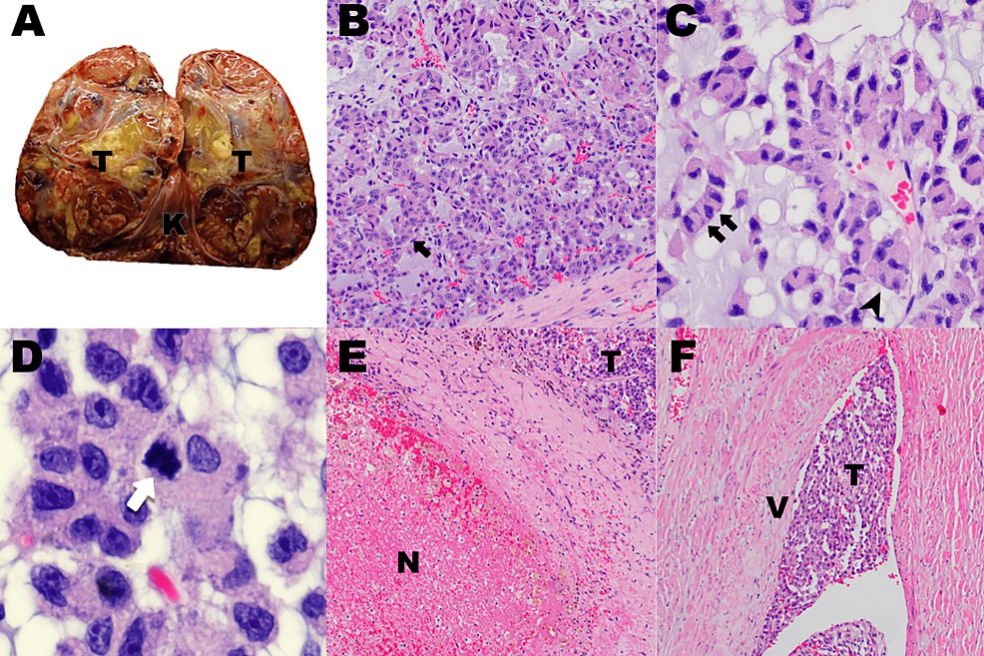
Macroscopic and microscopic images of the tumor. (A) Bivalved nephrectomy specimen with suprarenal mass. (B) Tumor cells arranged in trabecular patterns (black arrow). (C) Tumor cells arranged in cords (double black arrow) and papillary patterns (arrow head) with neoplastic cells exhibiting hyperchromatic nuclei with occasionally conspicuous nucleoli, and abundant eosinophilic cytoplasm. (D) Rare mitotic figure (white arrow). (E) Tumor with necrotic debris. (F) Vascular invasion K: kidney; T: tumor; N: necrosis; V: vein

Upon sectioning of the nephrectomy specimen, a well-circumscribed, yellow to tan, variegated with translucent areas, soft mass measuring 20.8 x 14.4 x 4.1 cm was found at the superior pole (Figure 2A). A thin fascia was seen demarcating the renal parenchyma from the tumor.

Histologic sections showed neoplastic cells arranged in trabecular (Figure 2B) and papillary patterns and cords (Figure 2C), surrounded by a myxoid background (Figure 2B-2C). These cells have occasionally enlarged, round to ovoid, hyperchromatic to vesicular nuclei, with inconspicuous to prominent nucleoli, and abundant eosinophilic cytoplasm (Figure 2C-2D). Rare mitotic figures were seen (Figure 2D). Necrotic debris (Figure 2E) and vascular invasion (Figure 2F) were identified.

**Figure 3:**
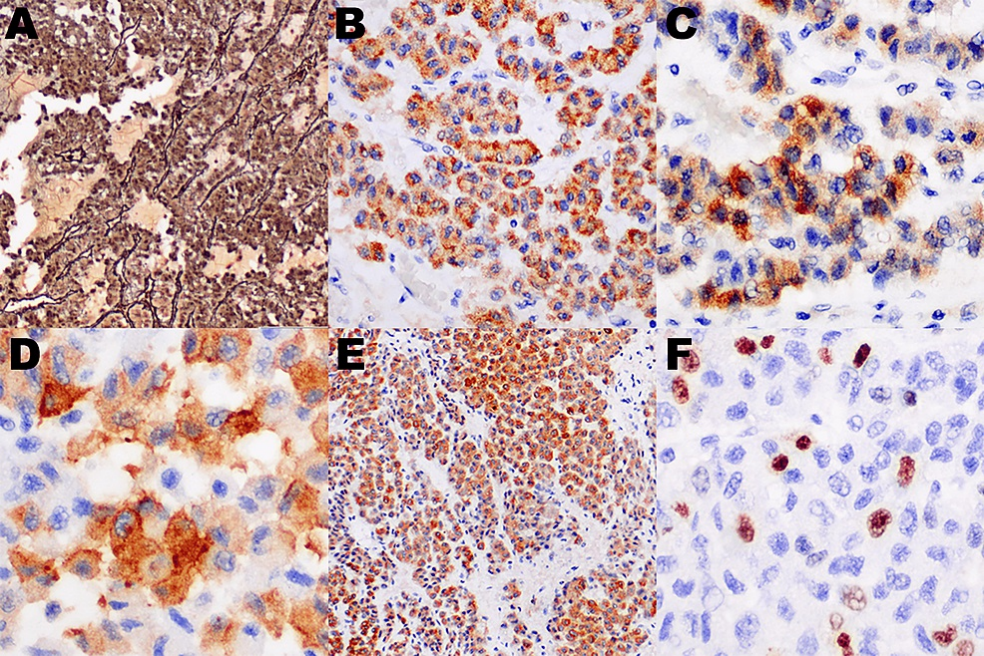
Special and immunohistochemical stains. (A) Reticulin stain, (B) Melan A, (C) Inhibin, (D) Synaptophysin, (E) Pancytokeratin (AE1/AE3), (F) Ki-67 (MIB1)

Assessment of the behavior of this tumor was done using the Weiss scoring system, modified Weiss system, reticulin algorithm, and Helsinki scoring system. Mitotic count of eight (8) per fifty (50) high power fields, necrosis, clear cells ≤ 25%, and venous invasion were noted, giving a Weiss and modified Weiss total score of four (4). An altered reticulin framework (Figure 3A) associated with a mitotic count of eight (8) per fifty (50) high power fields, tumor necrosis, and vascular invasion is indicative of a malignant behavior according to the reticulin algorithm. Table 1 summarizes the histopathologic findings and scoring system criteria that were present in the tumor.

**Table 1:** Summary of the histopathologic findings using the different multiparameter scoring algorithms for ACC

Scoring system	Criteria present in the tumor	Score	Total
Weiss scoring system	Mitotic count >5 per 10mm^2^ (50 high-power fields)	1	4
	Necrosis	1	
	Clear cells ≤25% of tumor volume	1	
	Venous invasion	1	
Modified Weiss system	Mitotic count >5 per 10mm^2^ (50 high-power fields)	2	4
	Necrosis	1	
	Clear cells ≤25% of tumor volume	1	
Helsinki scoring system	Mitotic count >5 per 10mm^2^ (50 high-power fields)	3	23
	Necrosis	5	
	Ki-67 proliferation index (%)	15	
Reticulin algorithm	Altered reticulin framework associated with mitotic count >5 per 10mm^2^ (50 high-power fields), tumor necrosis, and vascular invasion	N/A	N/A

Immunohistochemical studies were performed to differentiate myxoid ACC from anaplastic lymphoma kinase-rearranged renal cell carcinoma (ALK-rearranged RCC). The neoplastic cells expressed Melan-A, Inhibin, Synaptophysin, and Pancytokeratin (Figure 3B-3E). Evaluation for Ki-67 proliferation index was done at 400x magnification by counting the total number of neoplastic cells with nuclear staining divided by the total number of tumor cells (500-2000 tumor cells) in areas with the highest density of positive neoplastic cells (hotspots). Mitotic activity of eight (8) per fifty (50) high power fields, tumor necrosis, and Ki-67 proliferation index of 15% (Figure 3F) warrant consideration of an ACC based on the Helsinki scoring system.

**Figure 4:**
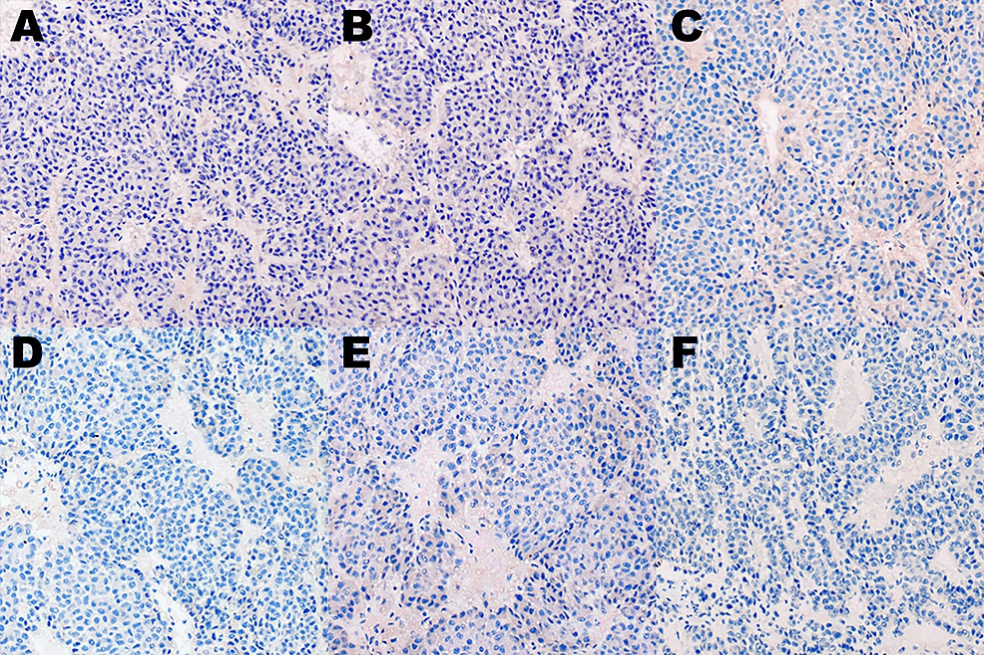
Immunohistochemical studies for (A) CK7, (B) CK20, (C) GATA-3, (D) PAX-8, (E) ALK, (F) CA IX CK: cytokeratin; ALK: anaplastic lymphoma kinase; CA IX: carbonic anhydrase IX

The tumor cells did not express CK7, CK20, GATA-3, PAX-8, ALK, and carbonic anhydrase IX (CA IX) (Figure 4). Negative expression for GATA-3 (Figure 4C) rules out a urothelial origin of the neoplasm, while negative expression for ALK and PAX-8 (Figure 4D-4E) rules out a diagnosis of ALK-rearranged RCC. Given the immuno-morphologic features of this tumor, a diagnosis of myxoid adrenal cortical carcinoma was rendered.

Post-operatively, the patient completed an initial six cycles of chemotherapy with carboplatin, etoposide, doxorubicin, and palonosetron. However, due to financial constraints, the patient decided to change her attending physician and have her chemotherapy at a nearby hospital. Currently, the patient is on chemotherapy with doxorubicin only.

## Discussion

Myxoid ACC is a rare subtype of ACC, with only around 42 reported cases in literature as of the year 2020 [1]. It commonly occurs in the fifth decade with no sex predilection [1]. The left adrenal gland is usually affected [1].

This aggressive neoplasm is characterized by a variably abundant myxoid stroma (5-90% tumor) and two recognizable morphologic patterns: (1) small neoplastic cells with mild atypia arranged in cords and microcysts, with notable myxoid background, and (2) neoplastic cells similar to a conventional ACC (i.e., large neoplastic cells with eosinophilic cytoplasm arranged in diffuse or nodular patterns) surrounded by focal myxoid changes [3]. ACC with a myxoid component of <20% is designated as conventional ACC with focal myxoid degenerative changes [3].

Different multiparameter algorithms (i.e., Weiss scoring system, modified Weiss system, Reticulin system, and Helsinki scoring system) (Table 1) [1-3] can be employed to establish the malignant behavior of an adrenocortical neoplasm. Both the Weiss and modified Weiss criteria need a total score of ≥3 to establish a diagnosis of ACC [2]. However, some parameters in the Weiss criteria (i.e., diffuse growth pattern, lymphatic invasion, nuclear atypia) may not be appreciated, and this might pose a diagnostic challenge in the diagnosis of myxoid ACC [1-2]. The reticulin algorithm states that for an adrenal neoplasm to be designated as malignant, the following criteria must be met: an altered reticulin framework associated with one of the following malignant features (i.e., mitotic count >5 per 10mm2, tumor necrosis, and vascular invasion) [2]. The Ki-67 proliferation index, necrosis, and mitotic activity are used in the Helsinki scoring system to indicate malignant behavior (score > 8.5) [2]. A Helsinki score of >17 portends a dismal clinical outcome [2]. It is important to note that due to the rarity of myxoid ACC, all of the accepted scoring algorithms can be used for histopathologic assessment [2].

**Table 2:** Different multiparameter scoring systems applicable only to the Conventional, Myxoid, and Sarcomatoid subtypes of ACC

Scoring system	Criteria present in the tumor	Score	Total score for malignancy
Weiss scoring system	High Fuhrman nuclear grade (III or IV)	1	≥3
	Mitotic count >5 per 10mm^2^ (50 high-power fields)	1	
	Atypical mitosis	1	
	Necrosis	1	
	Diffuse architecture >30% of tumor volume	1	
	Clear cells ≤25% of the tumor volume	1	
	Capsular invasion	1	
	Venous invasion	1	
	Sinusoidal (lymphatic) invasion	1	
Modified Weiss system	Mitotic count >5 per 10mm^2^ (50 high-power fields)	2	≥3
	Clear cells ≤25% of the tumor volume	2	
	Atypical mitosis	1	
	Necrosis	1	
	Capsular invasion	1	
Helsinki scoring system	Mitotic count >5 per 10mm^2^ (50 high-power fields)	3	> 8.5
	Necrosis	5	
	Ki-67 proliferation index (%)	Numeric value of Ki-67 from the hotspot	
Reticulin algorithm	Altered reticulin framework associated with mitotic count >5 per 10mm^2 ^(50 high-power fields), tumor necrosis, and vascular invasion	N/A	N/A

Both mitotic activity and the Ki-67 proliferation index have prognostic significance in ACC [2]. A Ki-67 proliferation index of >5% is suggestive of the malignant behavior of the tumor [2]. ACC are traditionally categorized into low-grade (≤20 mitoses per 50 high-power fields) and high-grade (>20 mitoses per 50 high-power fields) categories based on the tumor’s mitotic activity [2].

The immunophenotype of myxoid ACC is similar to that of a conventional ACC [1-4]. Hence, the diagnosis hinges more on the histomorphology of the tumor. Given the location of the tumor and its myxoid features, a possible differential diagnosis for a myxoid ACC is an ALK-rearranged RCC [5-6]. In this case, immunohistochemical studies pose valuable tools to determine the origin of the tumor (Table 1). Cytokeratin markers such as Pancytokeratin (AE1/AE3), CK7, and CK20 can help determine the primary site of the tumor. Positive expression for Pancytokeratin is indicative of an epithelial nature of the tumor. Given the tumor site for the case, a negative expression for CK7 and CK20 suggests that the primary site of the tumor may be renal or adrenal. PAX-8 and GATA-3 are immunohistochemical stains used to establish a renal or urothelial nature of the tumor. Positive expressions for Inhibin, Melan-A, and Synaptophysin are specific for an adrenocortical origin [1-2]. ALK is done specifically for ALK-rearranged RCC [5-6].

**Table 3:** Difference between myxoid ACC and ALK-rearranged RCC ACC: adrenocortical carcinoma; RCC: renal cell carcinoma; PanCK: Pancytokeratin; CK: cytokeratin; ALK: anaplastic lymphoma kinase; CA IX: carbonic anhydrase IX. Reactivity: +: positive; -: negative; ± variable

Immunohistochemical stains	Myxoid ACC	ALK-rearranged RCC	Urothelial carcinoma
PanCK AE1/AE3	±	+	+
Inhibin	+	-	-
Melan-A	+	-	-
Synaptophysin	+	-	-
CK7	-	-	+
CK20	-	-	+
GATA-3	-	-	+
PAX-8	-	+	-
ALK	-	+	-
CA IX	-	-	-

Distant metastasis is a distinct feature of this neoplasm, with the lung and the liver as common sites of metastasis [1]. The median survival of individuals with myxoid ACC is 29 months [1]. While surgical intervention is a mainstay in the treatment of adrenal cortical carcinoma, there are no clear guidelines on the management of myxoid ACC [4].

## Conclusions

Myxoid ACC is a unique ACC that may be missed due to its lack of atypia and diffuse growth pattern. An exhaustive sampling of the tumor is required in order to differentiate myxoid ACC from conventional ACC with focal myxoid degenerative changes. Appropriate usage of the known multiparameter scoring systems and immunohistochemical studies is encouraged to prevent the underrecognition of this malignancy. Furthermore, additional studies are recommended to provide the most appropriate criteria for histopathologic diagnosis and to establish therapeutic approaches for this aggressive neoplasm.
